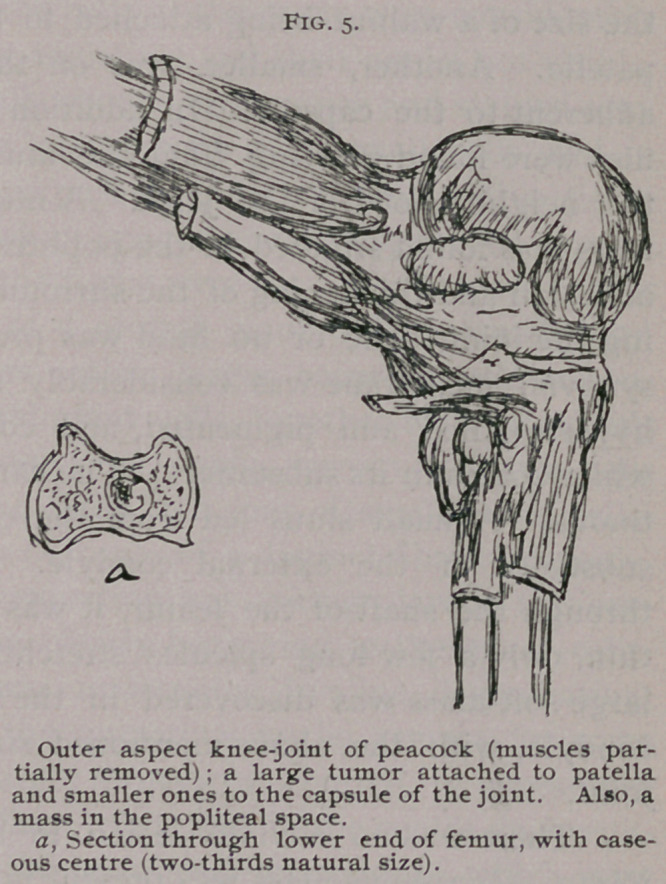# Tuberculosis in Birds

**Published:** 1890-06

**Authors:** Walter K. Sibley

**Affiliations:** B.A., M.B., B.C., Cambridge; M.R.C.S., England


### THE JOURNAL
OF
### COMPARATIVE MEDICINE AND VETERINARY ARCHIVES.

VOL. XI. JUNE, 1890. NO. 6.

### TUBERCULOSIS IN BIRDS.

BY WALTER K. SIBLEY, B.A., M.B., B.C., CAMBRIDGE; M.R.C.S., ENGLAND.

IT is proposed, in this article, to describe the disease known as tuberculosis as it is found in birds. Although many examples have been observed in various members of the avian class, a considerable number of cases examined occurred in the domestic fowl. The general description given may be considered as referring to fowls, unless another species of bird is mentioned. The abdominal organs, that is, the spleen, liver and intestine, were the parts primarily affected. Rarely was there any fluid in the peritoneal cavity, but the abdomen was often bulged. On opening it the liver, from its enormous size, appeared to occupy the greater part of the cavity and presented yellowish deposits on the surface and in the substance of the organ of various sizes. The spleen was likewise usually enlarged many times its natural size. In some cases no obvious deposit was seen in the intestines; at other times these organs were beset with hard nodules, often as large as beans. The *liver* was usually studded with deposits varying in size from a millet seed to that of a pea, the organ itself being excessively friable and the capsule much thickened. Sometimes only one segment of the liver contained deposits, which in some instances were very advanced in degenerative changes, the remainder of the organ being apparently healthy. In a very advanced stage the whole organ presented much the appearance of a piece of "almond rock," being a mass of caseous nodules loosely held together by fibrous tissue.

Microscopically, in an early stage, circular areas of epithelioid cells were seen, apparently lying in vessels and enclosed by their walls. Sections in a more advanced stage showed a central region formed by clumps of tissue, some being caseous and others hyaline. These often appeared to be giant cells undergoing changes, in places still revealing their many nuclei. The central masses of caseation being immediately surrounded by a zone of cells ill-defined and deeply stained, often of a hyaline nature. Between these, clear spaces were seen. The rest of the deposit was formed by granulation tissue, the whole usually very definitely encapsuled by fibrous tissue. Secondary areas of degeneration often occurred in the outer zone of small cells and in the fibrous capsule. In a more advanced deposit, each area consisted of an irregular central mass of caseation extending in the form of pseudopodia into the next area; bacilli were present, usually in groups, toward the periphery of this degenerated tissue. Between these processes of progressive degeneration large cells (apparently giant cells), were seen scattered among the small round cells. Cells of an epithelioid nature also occurred in this zone of granulation tissue, the whole surrounded by a capsule of fibrous tissue. Occasionally there was some infiltration of leucocytes among the liver cells around. In a still more advanced stage where many deposits occurred together, there was a considerable development of myxomatous tissue around them, producing a good deal of new tissue formation, in which again were seen smaller deposits. The caseous material was often stained yellow, as if by bile, and the same coloration was sometimes seen in all the degenerated deposits throughout the body. In a few examples of apparently a very chronic nature nearly the whole liver was transformed into very definite areas, consisting of caseation lying in distinct partitions of dense fibrous tissue (well seen with the naked eye), thus leaving but little original hepatic tissue to carry out the functions of the organ. The gland, or large segments of it in this stage, consisted of dense bundles of fibrous tissue, in some places of a myxomatous nature, and in other parts of a dense fibrous tissue, more or less infiltrated with small cells. Scattered about in this new tissue were small islands of caseation which appeared to be gradually undergoing more and more compression by the fibrous tissue growth around, until those areas near the surface of the organ were completely separated, and so a spontaneous cure of that part occurred. At times, immediately surrounding the caseous foci, the remnants of epithelioid cells, or of giant cells, were seen ; at other times the caseation was immediately enclosed by the fibrous tissue, here and there often infiltrated by small round cells, all traces of epithelioid cells and other elements of the original tubercles being amalgamated into the caseous centres. In an organ in this condition large segments, or even the greater part of the whole organ, were devoid of liver cells, or at the most simply showed a few isolated and much changed liver cells lying in the bundles of fibrous tissue. In some more peripheral parts infiltrated groups of hepatic secreting cells might be seen in places. As to the exact seat of the deposits, it was in the vessels, probably in the lymphatics, and the original vessel wall appeared to give rise by proliferation and subsequent degeneration to the tubercular elements.

The arrangement of the bacilli in such a case was also of interest, in that practically none were present in the new-formed fibrous tissue ; but in the caseous centres, bacilli occurred in densely packed groups, not evenly scattered throughout the degenerated mass, nor were the individual groups very numerous. In those centres which were apparently of oldest formation and most advanced in degeneration the bacilli were completely absent (obsolete tubercle).

The *spleen* was usually enormously enlarged, and then but little splenic tissue remained, the whole being a mass of caseous material. Microscopically, the deposits were seen more or less grouped together in fours or fives, each group being again distinctly encapsuled by fibrous tissue. In the centre of each caseous matter, in fairly large clumps, were seen giant cells, these being immediately surrounded by a deeply stained zone of ill-defined large cells, followed by small round cells and fibrous tissue. Often there was considerable small-celled infiltration into the tissues round these deposits, together with, in places, small roundish groups of granular epithelioid cells. Although the deposits occurred several together, isolated ones of the same nature were occasionally seen. A few groups of epithelioid cells also occurred scattered in the splenic tissues.

The *pancreas* was very rarely affected, and when it was a few caseous deposits were found in its centre, apparently in connection with the vessels. In the case, however, of a crane with very advanced disease, all the intestines and the other abdominal viscera were bound together and to the abdominal walls by caseous masses, and in this example the pancreas likewise was almost completely caseous, but little glandular tissue remaining. In fact, here all the hollow abdominal viscera appeared imbedded in

FIG. 1.

FIG. 2.

1. Piece of small intestine with large nodules (one-half natural size).

2. Intestine at junction of cæca, showing considerable deposit in wall of latter (one-half natural size).

In. Intestine ; r. Rectum ; cc. Cæca.

a solid mass of caseous matter, section through which revealed the cut ends of the various folds of the intestine.

In the *intestines*, small areas of infiltration appear in the mucosa, consisting of leucocytes, with here and there nests of epithelioid cells, the process extending into the muscular layer, and the whole wall became infiltrated by groups of epithelioid cells and leucocytes. The centres of the larger collections of epithelioid cells soon degenerate and become caseous, the mucous membrane over them gives way, a small ulcer with thickened edges is formed, with at first a very minute aperture into the bowel. The deposits then break down more rapidly than the mucous membrane, which thus becomes undermined, and so a sacculated cavity, extending on the outside of the gut and filled with debris is formed. Its walls go on increasing from fresh deposits of tubercle together with thickening of the serous coat. From time to time the debris from the centre of these is discharged into the lumen of the digestive tract, and thus an enormous quantity of caseated material is removed from the system. In the above way caseating masses or pouches were produced, varying in size from a split pea to a marble, or even in one case to that of a hen's egg. (Figs. 1, 2 and 3.) These masses, though more prominent externally, yet cause considerable mechanical obstruction to the progression to the intestinal contents; so the bowel becomes dilated above each of these, giving the whole digestive tract a very irregular appearance. In the cases in which the cæca were affected, the chief deposit was at their junction with the main gut, and so they become much dilated, especially at their extremities, which, in some cases, contained entozoa (ascaris vesicularis). (Fig. 2.) Frequently the œsophagus, or the proventriculus, presented lesions having the same characters, and in some cases there were circular ulcers in the gizzard.

FIG. 3.

Showing sacculated pouch from wall of proventriculus (one-half natural size).

Œ, Œsophagus; Cr, Proventriculus; Gi, Gizzard; Duo, Duodenum; nn, Nodules.

The instance of a vulture presented almost a complete ring of cicatricial tissue from old ulceration at the junction of the œsophagus and stomach. This showed several papillæ, formed apparently by adhesions of the thickened sides of old ulcers after the complete ejection of their contents into the alimentary canal. In a fowl a large pouch was found from the posterior wall of the proventriculus. This pouch, the size of a pigeon's egg and resembling a sacculated aneurism (Fig. 1), was filled with debris from caseation and partly digested food stuff. Its walls were composed of caseous material and fibrous tissue ; it communicated with the proventriculus by a small, nearly circular opening, about half a centimetre in diameter. A second smaller sac about the size of a marble was present, a little higher up, projecting from the anterior wall of the œsophagus. Under the microscope a section through the wall of this sac showed it to be composed of an external layer of fibrous tissue in places caseous, and becoming more so at the internal surface, where it appeared to be crumbling away. In the deeper parts of the fibrous tissue were large spaces filled with small round cells. There was considerable small-celled infiltration at the junction of this with the metamorphosed tissue, together with a line of degenerated giant cells. Enormous numbers of bacilli were scattered both in groups and singly toward the internal surface of the degenerated tissue, but few were found in the deeper regions, or at the junction with the fibrous tissue, where the process of decay was most active.

In one instance an irregular caseous node was seen developed from the lower and anterior part of the proventriculus and extended into the walls of the gizzard. This tumor had produced an extensive depression in the liver, where it was in contact with this organ. In another case the abdominal cavity was occupied by a large bilobed cyst, which arose from one of the cæca. The walls of this sac were adherent to the posterior body wall and to all the abdominal viscera in contact with it. The walls of the larger lobes of the cyst were exceedingly thin, the other smaller lobe, that is, immediately adjoining the one in communication with the cæca, had its walls much thickened and presented much the appearance of the sac described above in another instance as arising from the proventriculus (Fig 3). The whole was filled by a flaky, milky fluid and lined to a varying extent by caseated material. All the mesenteric vessels were much dilated and filled with blood there was no obvious deposit in the other cæca nor in the rest of the gut. The liver and the spleen contained small deposits. Sections of the wall of this cyst showed the presence of numerous bacilli arranged much as in the one above described as springing from the proventriculus.

In all cases in which the gut was affected, the mesentery was more or less infiltrated with small cells, especially along the line of the bloodvessels, and in many places circumscribed areas of leucocytes were seen ; in some places completely surrounding, and in others on the side of small capillaries, more advanced stages with central degeneration were occasionally found.

In many cases the *lungs* presented no obvious lesion ; in a few there were slight pleural adhesions, usually posteriorly. The most frequent lesion occurred in the form of a small isolated nodule the size of a split pea on the anterior surface of the lung toward its base. Upon section these were of a fibro-cartilaginous nature and usually close to the surface and covered by thickened pleura ; in no case was there any distinct deposit at the apex, or any cavity formed in the substance of the lung. In one specimen nearly the whole of one lung and the base of the other were caseating masses, but this appeared to be secondarily by direct extension from caries of the adjacent vertebræ. Here the process had also extended up the bronchus into the trachea, and up this into the syrinx. The deposit was chiefly in the adenoid tissue posteriorly, and visible upon opening up the trachea as small, pale projections beneath the mucous membrane along the posterior border. This diminished in quantity as one proceeded from below upward, very abundant and almost surrounding the bronchus of this side ; it gradually decreased until the syrinx was reached, in which recent deposits only were present, those lower down having undergone caseation.

Microscopically, the whole lung was often seen to be more or less infiltrated with small cells, and in some cases circumscribed areas of deposit leading to nodules of caseation were seen with thickened pleura over them. A section through one of the more recent nodules showed great infiltration of the peribronchial regions with small cells. In one case in the centre of some of these smaller infiltrated bronchioles a fungus (saccharomyces mycoderma) was present. Also, microscopically, a section from about the middle of the trachea showed much submucous infiltration posteriorly with scattered nodules of caseation containing large numbers of bacilli.

In the *heart* of some cases considerable thickening of a myxomatous nature of the mitral valve was found. In a few cases there were caseating lymphatic glands in the lower and deeper tissues of the sides of the neck, but in none had they suppurated or discharged externally, nor did there appear to be much infiltration of the tissues around.

Usually the *kidneys* and *ovary* were normal ; in a few there was some deposit about the hilus of both, and occasionally the disease had penetrated into the substance of the organ, but in no case was there miliary deposit in the kidney itself. In two cases, in both of which the visceral lesions were in an early stage, some caries of the vertebræ was present, and in one also hip-joint disease, with an old cavity in the upper part of the thigh bone.

A case of disease of the spinal column presented in the abdominal cavity a large abscess sac containing some two ounces of flaky fluid, extending on the left side posteriorly down into the pelvis and displacing the spleen and liver. The spleen was raised forward and firmly adherent, being almost imbedded in its wall ; the left lobe of the liver was also adherent to the anterior surface, and the kidney of this side to the posterior surface. Upon opening up this sac its walls were found to be thin and very friable, covered internally by an irregular deposit of flaky material, and containing lymph-like pus with solid bodies suspended in it. This main sac communicated through the rudimentary diaphragm by a narrow passage with a small cavity in the base of the left lung pleura (the pleura, lung and diaphragm being one cheesy mass). The whole lung of this side, with the exception of the apex, was solid and adherent to the spine, upon separation from which some caries of the bodies in the dorso-lumbar region was exposed, extending over some four or five vertebræ. The abscess appeared to originate from this, pus channels being traced to the diseased bone. There was also some caseation of the opposite lung with fluid, its pleura, in connection with the spinal caries and pus channels, going through to the other side.

In another case there was hip-joint disease on the left side, which was chiefly confined to the acetabulum, the floor of which was replaced by fungating masses of granulation tissue projecting into the interior of the pelvis ; there was little or no fluid in the joint, but considerable matting together of the tissues in the neighborhood. The head of the femur was movable and had slight erosion of its cartilage. The thigh-bone readily fractured through its neck, and it was then found that in the region of the tuberosities was a cavity filled with a very dry nodule of caseated material the size of a large pea, in which tubercle bacilli were present. The walls of this cavity were quite smooth and apparently of old standing, the caseated nodule being firmly encapsuled by the surrounding bone.

In the region of the lower dorsal spine was slight erosion of the bodies of the adjacent vertebræ, the right pleura being filled with flaky, blood-stained pus, and in close contact with the diseased bone. The lung itself was healthy ; no fluid was in the opposite pleura.

Microscopically, a section through the neck of the femur showed some of the so-called air cavities to be filled with small

FIG. 4.

Peacock ; tubercular tumors growing from ribs (two-thirds natural size).

FIG. 5.

Outer aspect knee-joint of peacock (muscles partially removed) ; a large tumor attached to patella and smaller ones to the capsule of the joint. Also, a mass in the popliteal space.

*a*, Section through lower end of femur, with caseous centre (two-thirds natural size).

cells, some of which in places were undergoing caseation ; numerous bacilli were present in these patches of degeneration. In a few cases nodular deposits were found in the ribs, usually prominent on the internal surface. The bone over these was often much expanded and thinned, and had sometimes become quite absorbed, exposing the deposit in the centre of the bone on the surface.

The following case of a peacock I describe at greater length, in that it presented several points of special interest, tuberculosis being associated with bone disease, and, a point specially worthy of note, with very extensive amyloid degeneration. The bird was extremely emaciated and presented several tumors of a caseous nature, often as large as walnuts, on various parts of the body. A large one was situated on the back, firmly attached to the inferior angle of the scapular. A second mass, not quite so large, was found between the plains of the muscles in this region. Another was perforating the vertical plate of the sternum toward its lower end. A mass of the same nature was seen on the inner surface of the ribs (Fig. 4), producing their complete absorption.

The right knee-joint (Fig. 5) was beset with large masses, one the size of a walnut being attached to the anterior surface of the patella. Another, smaller, was on the inner side of the joint adherent to the capsule. In addition to these, many small nodules were found growing from the muscular tendons inserted in the neighborhood of the joint. A nodule with a loose central mass was found situated in the popliteal space, with considerable adhesion and thickening of the surrounding tissues. Upon opening the joint, little or no fluid was present in the interior. The synovial membrane was considerably metamorphosed, in places hypertrophied and pigmented, and contained numbers of small white bodies in its substance. The cartilages were soft and gelatinous. A small sinus led from the cavity of the joint into the substance of the external condyle. Upon making a section through the shaft of the femur, it was found to be exceedingly thin, only a few long spicules stretching across its interior. A large soft mass was discovered in the substance of the external condyle, with the above-mentioned sinus connecting it with the joint.

There was no obvious lesion to be discovered in the other joints. A caseous mass was present in the right side of the neck, distinct and free from the œsophagus or the trachea. A deposit the size of a pea occurred on the external surface of the pericardium. Another caseous mass was found projecting from the walls of the superior vena cava immediately above its entrance into the pericardium, and separated from the lumen of the vessel by a thin membrane. Upon opening the heart considerable thickening of the mitral valve was seen. The liver was somewhat enlarged, its surface smooth, having a pale and waxy appearance upon section. Its substance was studded with yellowish-white miliary deposits. The spleen was also enlarged, and contained deposits which were larger than those seen in the liver. The kidneys were pale, surface slightly irregular and exhibited a few small deposits in their substance.

The digestive tract was considerably affected with nodular enlargement of its walls ; one nodule occurred in the œsophagus, several in the duodenum, while isolated deposits were seen in the mesentery. The cæca, contrary to what was usually found in fowls, were free from deposits, they being abnormally full of fæces.

*Microscopical Appearances.*

In the liver, in parts, the cells of the organ were considerably altered, their outlines being indistinct and their nuclei not visible. The whole tissue had a dull, opaque appearance and stained very imperfectly. Between the lobules were a few small cells, and in places vessels filled with corpuscles. All the connective tissue and walls of the vessels had an opaque and homogeneous appearance. Scattered through the tissue were some small, more or less round deposits, consisting of a central giant cell surrounded by granulation tissue. Others rather larger occurred, which consisted of one or more central giant cells, together with large cells with indistinct nuclei ; these were, again, surrounded with cells of an epithelioid nature, and these by a zone of small round cells. The whole granuloma was destitute of a distinct fibrous capsule. In most of these some degeneration was taking place in the central regions and often was clearly beginning in the giant cells, the protoplasm of which was undergoing hyaline degeneration, in the substance of which the numerous nuclei remain for some time distinctly visible. Larger structures occurred, in appearance as thrombosed vessels. These contained in the centre an irregular hyaline mass, between the peripheral processes of which were numbers of giant cells. Surrounding these was a zone of granulation tissue, the granuloma being enclosed as if by a thickened vessel wall, on the inner surface of which more giant cells were sometimes seen. It would seem that various stages, from the small deposits above mentioned to these apparently thrombosed vessels, can be traced with epithelioid and sometimes giant cells, the whole appearing to be produced from a proliferating endothelium. The endothelial lining, however, of most of the vessels, as the portal vein, hepatic artery and bile ducts, appeared normal. With the iodine solution the connective tissue and walls of the vessels, together with many of the liver cells, gave the mahogany-brown color, which deepened on the addition of sulphuric acid. With methyline violet the same regions stained red, the various deposits staining blue. No bacilli could be found in any of the deposits in the liver.

The splenic tissue was profoundly altered, the walls of the vessels being considerably thickened, and these were in some cases thrombosed. On examining more minutely one of these thrombi it was seen that the original vessel wall existed as a fibrous capsule, containing in places an artery with thickened walls. Within this was a broad zone of granulation tissue, with a few vessels and epithelioid cells, with here and there an irregular patch of hyaline degeneration. Nearer the centre a zone of flattened epithelioid cells presented itself, within which, again, was a ring of giant cells, the centre of the whole being formed by a mass of degenerated tissues, in which sometimes tubercle bacilli occurred. The whole of the splenic pulp and the bloodvessels gave the amyloid reactions.

In the kidney the cells of the tubules appeared to be normal. Many of the glomeruli had undergone change and presented an opaque appearance. A few thrombosed vessels were present. With the iodine solution all the bloodvessels, both those in the glomeruli and those of the tubules, gave the amyloid reactions. With methyline violet it was seen that the amyloid change had affected especially the bloodvessels and the glomeruli, and to a less extent the connective tissues of the tubules.

In the heart sections of the mitral valve presented considerable myxomatous tissue formation in its substance.

The pericardial tumor consisted of a central caseous necrosis surrounded by a broad zone of tissue much infiltrated with small cells. Around the circumference of the necrotic tissue were numbers of giant cells. In this broad zone were numbers of groups of nests of epithelioid cells. Giant cells were also scattered irregularly about, the rest of the nodule being made up of small cells and fibrous tissue, in which hyaline globules were occasionally present. A few bloodvessels with changed walls were present in parts of the growth. Bacilli were seen in places in the caseous centres, but did not appear to exist in the tissue outside these. The substance of the larger tumors attached to the fibrous tissue around the bones and between the muscles was formed by a tissue of the same constitution, but with several foci of caseous degeneration of various stages, each centre having usually several giant cells immediately surrounding it. Bacilli did not appear to be present either in the giant cells or in the epithelioid cell nests and only sparingly in the clearer parts of the same caseous material. The mass in the popliteal space was formed of the same sort of tissue, but contained many more large cells and hyaline globules, and in its centre was a small mass of cells like that of a lymphatic tissue. No tubercle bacilli were found in this nodule. The soft mass in the centre of the substance of the external condyle consisted of a collection of granulation tissue, which had in places undergone caseous and hyaline degeneration. A few epithelioid cells were also present, also some giant cells scattered about, together with many albuminous and fatty globules. Bloodvessels filled with corpuscles were also numerous.

Sections through the synovial membrane of diseased knee-joint showed the serous membrane to be much metamorphosed. The endothelial cells appeared to be replaced by a granulation tissue, the cells of which were much swollen, forming globules which gave the amyloid reactions. On the surface many granulomata were seen, some of which were undergoing central degeneration. These are the small white bodies which were noticed in the description of the macroscopic appearances of the joint.

In a swan examined, the walls of the large abdominal air-sacs were lined by small caseous deposits, especially abundant on the part attached to the lower ribs, and from the walls of these air-sacs a complete chain of nodules extended to the liver along the inner surface of the abdomen, produced by a complete thrombosis and caseation of the large lymphatic vessels, both those in the mesentery and also those along the posterior abdominal wall being thrombosed. This specimen also presented several ulcers in the gizzard, apparently of old standing, together with miliary deposits in the liver, spleen and inferior margins of the lungs. The right shoulder-joint also presented some thickening and matting together of the tissues around, with some fluid in the joint itself.

In some cases, especially in carnivorous birds, as owls, vultures, etc., the tubercular deposits in the viscera appeared to have a slightly different structure to the description given for graminivorous forms, in that the deposits in the viscera were very much more diffused and extensive. The whole substance of the liver or spleen in a comparatively early stage would be densely infiltrated with small round or oval deposits consisting of groups of epithelioid cells encapsuled by fibrous tissue, and these cells were all very full of tubercle bacilli, giant cells not being demonstrable. In later stages, after some degeneration of these epithelioid cells, giant cells were seen. Frequently in this stage the deposits were clearly within the vessels of the organ. So the intestinal deposits consisted, likewise, of groups or columns of epithelioid cells, extending through the serous and up through the muscular coat, and producing small tumor-like formations in the substance of the mucous membrane. These in a later stage would be found degenerated and caseous, leaving an ulcer with thickened edges. In fact, the deposits in these cases very closely resembled those described as leprous bodies in man.

Bacilli which both structurally [1] and in their reaction to staining fluids were apparently similar to those of tubercle bacilli found in man, occurred in great numbers in all the deposits, especially in those in which some degenerative changes had taken place. Although in great numbers in these regions, they were not found in cells outside these. From this it might appear that the bacilli were the result, and not the cause, of the morbid appearances, and that they were produced and grew in the products of degeneration. They soon absorbed the essential elements from the soil in which they grew, and so disappeared from the older regions of caseation and abounded in those of more recent formation. Hence they occurred toward the periphery of the caseous nodules in the viscera and toward the free edge of the pouch from the crop, and not in the deeper parts where the process of gradual metamorphosis of living tissue into a condition of coagulative necrosis was going on, and also were not present in deposits of old standing.

It would seem that the tissues of the avian tribe offer a soil in which tubercle develops to a much more extensive degree than in the human subject, and that the life energy of a bird is greater,

[1] The size and number of the bacilli varies in different species of birds, the difference probably being due to slight variations of the tissues (soil) of these animals one from the other.

withstanding a more prolonged strain upon its molecular constitution before the dissolution of the individual. So the collections of cells degenerate, the caseated products becoming irregular in outline, with bacilli present in great numbers, usually in groups toward their periphery. Structurally, the masses of caseation were very firm; in some cases a small furrow had appeared around them, and so they tended to become separated from the tissues in which they were formed, as seen on the surface of the liver. In some birds [pheasants] I have found these small, caseated nodules formed in the liver lying free in the abdominal cavity, and others on the surface of the liver in the process of separation. The deposits appear never to go beyond the stage of caseation; they do not calcify, and very rarely break down and form cavities. There is a great tendency to spread by continuity, as in the instance of the disease spreading from the spine to the pleura, lung and bronchus, and then up the trachea to the syrinx. Not only is the caseation very extensive, but there is frequently considerable new-tissue formation surrounding the nodules of degeneration; so the nodules are large, owing to considerable new formation of fibro-myxomatous tissue; in fact, the disease in this respect closely approaches a neoplasm in its nature.

The cases of the fowls were very interesting from an ætiological point, the disease being unknown in the other poultry yards in the neighborhood where the birds lived, many people about stating that they had never lost an adult bird from disease. The hygienic conditions of the locality were very good, being on the South side of chalk downs. The fowls were fed in a large field, and their huts water-tight and well ventilated. The tubercular disease in these cases appears to have been limited to certain broods; others which had lived for some years along with these—that is to say, with the same environment—remained exempt from the affection, as also pigeons living on the same farm never became subject to the disease. Again, wild birds, such as sparrows and starlings, feeding in the same poultry yard, were, from time to time, shot and examined, but in no case could any tubercle be found, although carefully watched for some six or seven years.

In these cases the disease appears to be a clear case of heredity, and must have been brought into the yard some years back, when the stock was originally started, and has transmitted itself with fatal regularity, in spite of every attempt to eradicate it by killing off and carefully getting rid of all affected birds. Here hygiene and good feeding have done little either to check the outbreaks of the disease or to stop it when it has once appeared in any given case. The only plan appears to be to kill off the whole stock and start afresh; for though frequently for some months no illness occurs, then, as soon as the birds arrive at a certain age, which appears to be associated with the reproductive processes—namely, young adult life—they one by one sicken, emaciate and die.

About three years ago some eggs for sitting were exchanged with some from a neighboring farm, and now this second farm, which was originally exempt, has lost several birds from disease. Here, again, the symptoms appear when the birds are between two and three years old, and, as in the original cases, the disease has so far not appeared in a male bird. [The original farm averages sixty adult hens and four cocks.]

Most writers on tuberculosis in fowls believe the disease to originate and to spread through the fowls consuming tubercle bacilli with their food, which in some cases was supposed to be traced in the first instance to the sputum of phthisical patients, and also that the original seat of the disease occurs in the intestine [Ribbert, De Lamallerée, Sutton, Nocard, Johne]. With these my observations do not permit me altogether to agree. In the first place healthy birds have not, as yet, been infected by tuberculosis by the direct experiment of feeding them on tubercle bacilli. Ribbert,[1] in Bonn, fed two hens on tubercular sputum for several months, and the autopsies showed only negative results. More recently, Straus and Wurtz,[2] in Paris, fed a cock and seven hens daily on tubercular sputum for a space of time varying from six months to a year. They state that some of the fowls in this time consumed as much as forty-five kilogrammes of sputum, and yet in not a single case was there the least evidence of tuberculosis after careful microscopical examination of the viscera. While in Germany I fed a pair of pigeons for two months on sputum rich in bacilli, and here, again, microscopical examination of the viscera revealed no trace of tubercle.

With respect to the primary seat of the disease I have carefully examined many cases of tuberculosis in different species of birds in which often both the liver and spleen showed abundant

[1] *Deutsch. Med. Wochenschr.*, 1883.
[2] Congres pour l'etude de la Tuberculose, 1888.

deposit, without being able to find any naked-eye lesion of any part of the intestine.

It must be added, however, that because no obvious lesion of the intestinal mucous membrane is observed, it does not by any means follow that the bacilli [if the cause of the disease] may not in these predisposed cases gain entrance to the system through this channel and produce considerable changes in the other abdominal viscera without leaving marked traces of their primary entrance. It appears that of all the organs which become affected with the disease the spleen stands first, not that I would maintain the spleen alone may be affected without the liver, but that it more rapidly reveals evidence of disease, seeing that often with but only microscopical deposit in the liver the spleen is enlarged many times its natural size and frequently completely caseous, leaving but little of the original splenic tissue to be seen. So, again, in cases of old-standing general tuberculosis such complete degeneration of the splenic tissues had occurred that no tubercle bacilli were to be found, they having all died out from want of nourishment. But in these cases bacilli continued to abound in the liver and some other regions of the body. I have thus far found tuberculosis in the following birds dying naturally in confinement, the diagnosis being in every case made from the microscopical appearance of granulomata undergoing central degeneration, together with the presence of tubercle bacilli : Canaries, doves, finches, fowls, geese, guan, owls, peafowls, pheasants, pigeons, swans and vultures.

From my observations I do not think that the disease occurs in a greater percentage of graminivorous than carnivorous species, but naturally many more opportunities arise for examining specimens from the former than from the latter class.

In concluding this paper and reviewing the description given, I must add that it appears that the disease known as avian tuberculosis in very many points more closely resembles leprosy than tuberculosis as generally understood occurring in man and some of the higher mammals.

The microscope reveals a somewhat remarkable analogy between tuberculosis as seen in birds and leprosy as described in man. Quoting from a recent contribution on leprosy we read: "Leprosy bacilli for a long time seem to be able to exercise comparatively little effect on the surrounding tissues and are able to multiply to an enormous extent without causing sufficient inflammatory action to lead to breaking down and ulceration." This, I may add, as far as it goes, is an exact description for the disease we are describing; for we have noted the extreme abundance of bacilli and the absence of breaking down of tissue even in the latter stages of the disease. So, also the close resemblance of the early deposits in various viscera to the leprous bodies, together with the clear presence of bacilli in the lymphatic vessels. Some have shown that the bacilli of leprosy are somewhat smaller than those of tubercle, and this appears to be the case with the bacilli of the disease in question, especially in fowls.

LITERATURE.

Larcher, *Recueil Vétérinaire*, 1871.
Crisp, *Trans. Path. Soc.*, London, 1872 and 1875.
Ribbert, *Deutsch. Med. Wochensch.*, 1883.
Johne, *Deut. Zeitschr. f. Thiermed.*, 1884.
Koch, *Mitth. a. d. Kaiserl. Gesundheitsamt*, 1884.
Sutton and Gibbes, *Trans. Path. Soc.*, London, 1884.
Cornil and Meguin, *Jour. de l'Anatomie et de la Phys.*, 1885.
Nocard, *Recueil de Méd. Vétérinaire*, 1885; *Brit. Med. Jour.*, 1886.
Klein, *Practitioner*, 1886.
De Lamellerée, *Gaz. Médicale*, 1886.
Sutton, *Journ. Comp. Med. and Surg.*, Philadelphia, 1886.
Sibley, *Trans. Path. Soc.*, London, 1888.
Straus et Wurtz, Congrès pour l'Etude de Tuberculose, Paris, 1888.
Moulé, Congrès pour l'Etude de Tuberculose, Paris, 1888.
Sibley, *Virchow's Archiv.*, 1889; *Journal of Anatomy and Physiology*, 1889.

## Figures and Tables

**Fig. 1. Fig. 2. f1:**
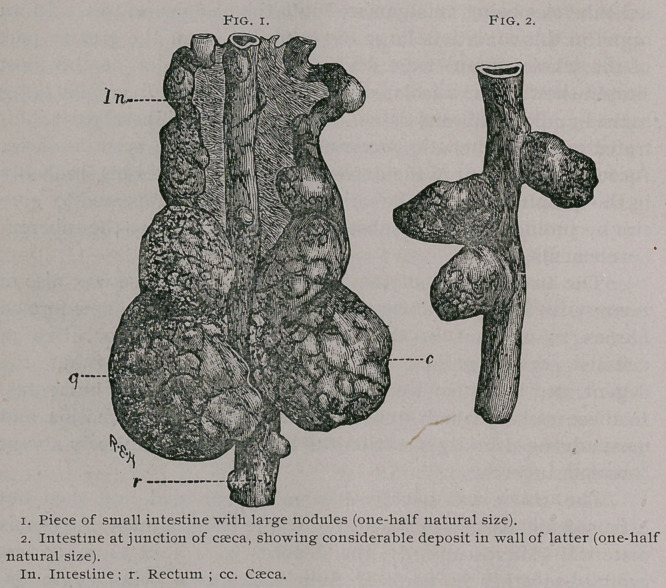


**Fig. 3. f2:**
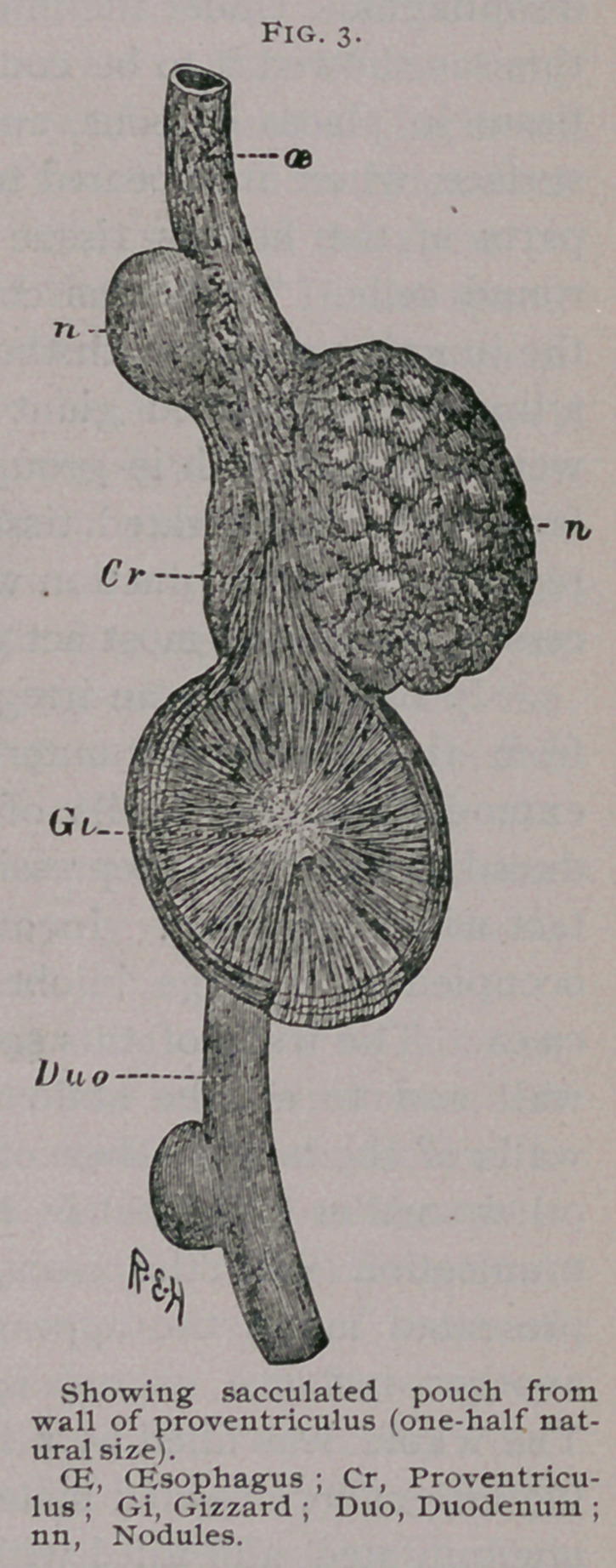


**Fig. 4. f3:**
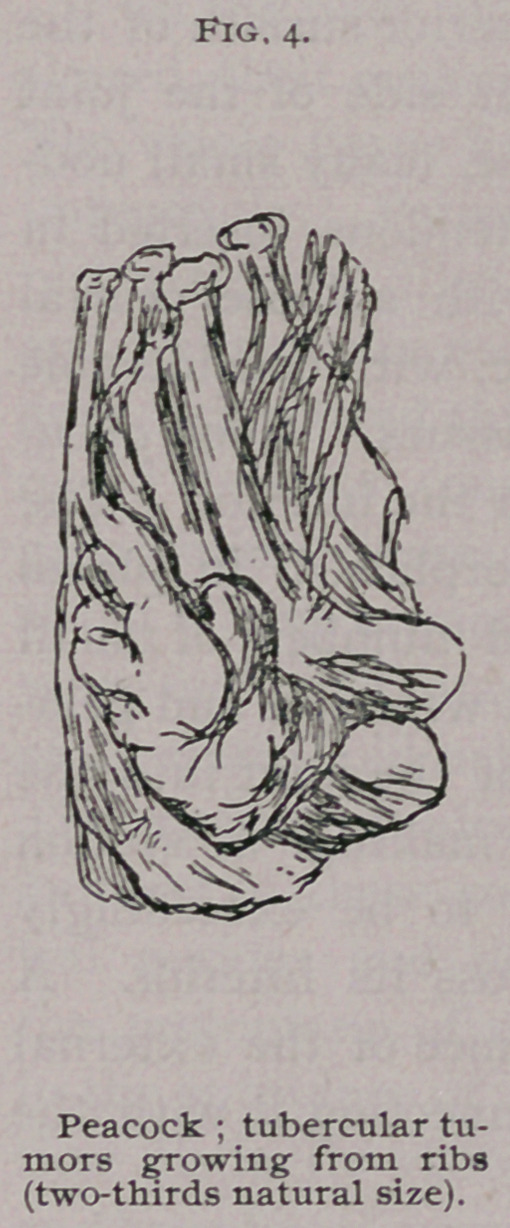


**Fig. 5. f4:**